# Analysis of local habitat selection and large-scale attraction/avoidance based on animal tracking data: is there a single best method?

**DOI:** 10.1186/s40462-021-00260-y

**Published:** 2021-04-23

**Authors:** Moritz Mercker, Philipp Schwemmer, Verena Peschko, Leonie Enners, Stefan Garthe

**Affiliations:** 1 Bionum GmbH - Consultants in Biostatistics, Hamburg, Finkenwerder Norderdeich 15 A, Hamburg, Germany; 2 Research and Technology Centre (FTZ) Kiel University, Hafentörn 1, Büsum, 25761 Germany; 3 Institute of Applied Mathematics (IAM) Heidelberg University, Im Neuenheimer Feld 205, Heidelberg, 69120 Germany

**Keywords:** Animal movement, Autocorrelation, Bio-logging, Habitat selection, Point process, Resource selection, Species distribution, Telemetry, Avoidance

## Abstract

**Background:**

New wildlife telemetry and tracking technologies have become available in the last decade, leading to a large increase in the volume and resolution of animal tracking data. These technical developments have been accompanied by various statistical tools aimed at analysing the data obtained by these methods.

**Methods:**

We used simulated habitat and tracking data to compare some of the different statistical methods frequently used to infer local resource selection and large-scale attraction/avoidance from tracking data. Notably, we compared spatial logistic regression models (SLRMs), spatio-temporal point process models (ST-PPMs), step selection models (SSMs), and integrated step selection models (iSSMs) and their interplay with habitat and animal movement properties in terms of statistical hypothesis testing.

**Results:**

We demonstrated that only iSSMs and ST-PPMs showed nominal type I error rates in all studied cases, whereas SSMs may slightly and SLRMs may frequently and strongly exceed these levels. iSSMs appeared to have on average a more robust and higher statistical power than ST-PPMs.

**Conclusions:**

Based on our results, we recommend the use of iSSMs to infer habitat selection or large-scale attraction/avoidance from animal tracking data. Further advantages over other approaches include short computation times, predictive capacity, and the possibility of deriving mechanistic movement models.

**Supplementary Information:**

The online version contains supplementary material available at (10.1186/s40462-021-00260-y).

## Introduction

The identification of factors that influence species distribution and resource selection is an important ecological issue [1] that has traditionally been addressed using appropriate regression methods based on presence-absence or count data [2–4]. However, recent technical developments involving radio- and telemetry-based approaches (e.g., Argos-, global positioning system-, or very-high frequency-based methods [5–8]) allow single-animal tracks to be recorded, thus providing a new and highly valuable alternative source of information to help answer such questions. These approaches allow the locations and movements of species to be analysed at a much finer spatio-temporal scale than previously possible.

Frequent ecological questions associated with animal tracks concern either the selection/avoidance of a certain resource/habitat or structure, or alternatively changes in behaviour related to such covariates. Both questions can again be applied at different spatial scales. On a small scale, the direct interaction of an animal with its surroundings can be investigated; e.g., by evaluating if certain habitats are used more intensively than others. On a large-scale however, certain areas or structures may assert effects on animal movement, for example as a result of direct perception (in an environment with no visual obstructions for ≥10 *km* [9]) or spatial memory [10, 11]. Animal movement is often shaped by a complex combination of all of the above-mentioned types of perceptions and memories interacting at various spatio-temporal scales [8, 12–14].

In contrast to count or presence–absence data where individuals are recorded relative to a discretised unit of area, tracking data only contain information about single points in time and space where the animal is present [15]. The statistical analysis of such ‘point observation data’ is often challenging, and various approaches have previously been developed and discussed (e.g., summarised in [8]). In summary, there are two distinctly different approaches for the analysis of animal tracking data.

The first approach is given by animal movement models that rely solely on tracking data points, e.g., by evaluating step sizes and turning angles. Variations on these models mainly differ from each other in the way in which time and space dependencies, as well as latent behavioural states, are entered into the model [8, 16–18]. Indeed, one of the main strengths of these approaches is the analysis of animal behaviour, possibly changing in relation to short-range or large-scale interactions with the environment. ‘Discrete-time hidden Markov models’ are a prominent and increasingly used example [17–21]. However, habitat selection cannot be directly assessed using such movement models, given that they lack quantification of habitat availability.

The second approach augments tracking points with an additional set of artificially created points (‘pseudo-absences’, ‘dummy points’, or ‘available steps’) to quantify habitat availability. The tracking points thus represent the used habitats, while dummy points are chosen optimally to measure/represent how much of each habitat type is available. This approach is frequently used in the context of spatial point observation data, and various sophisticated strategies have been used to select appropriate pseudo-absences (e.g., [22, 23]), frequently followed by an analysis using spatial regression or machine learning techniques, such as MAXENT or spatial logistic regression models (SLRMs) (e.g., [24–27]).

In the context of animal tracking data, such ‘dummy point’ approaches must however be used with care: first, the artificial generation of pseudo-absence points is an ad hoc approach and thus associated with several statistical disadvantages and criticisms: e.g., the choice of location and number of these points are often not straightforward, even though the regression results may sensitively depend on these choices [26–28]. Second, strong spatial, temporal, and angular autocorrelations are frequent challenges presented by tracking data [29, 30]: a tracking point is most likely to appear in spatial and temporal proximity to the previous point, and turning angles near zero indicate directional persistence. Furthermore, a correlation between non-zero turning angles indicates that animals are continuing to move in a similar way, such as circling birds. The importance of taking account of autocorrelations increases with the sampling frequency relative to the velocity of the animal [8], and neglecting these issues may lead to a distinct underestimation of parameter uncertainties [31, 32]. Appropriately accounting for autocorrelation in the context of ecological modelling is however complex: e.g., when considering spatial autocorrelation based on mixed modelling, fixed and random spatially varying covariates may be collinear (‘spatial confounding’), potentially causing biased interference [33–35], especially if covariates and residual structures act on similar spatial scales [36].

Two new methods have become available during the last decade, providing a rational basis for the choice of dummy points on the one hand, and integrating appropriate correlation structures on the other.

First, point process models (PPMs) naturally and automatically resolve many of the questions and pitfalls arising from previous techniques [8, 26, 27, 37]. In particular, PPMs allow the role and number of dummy points to be deduced purely mathematically by aiming at an efficient estimation of an integral as a part of the PPM likelihood [26, 37] leading to parameter convergence for sufficiently large dummy point numbers [38]. Additionally, PPMs represent a generalisation of many other frequently used methods [26, 39, 40], and the PPM likelihood can be approximated using various standard generalised linear modelling regression software [27, 41, 42] (possibly including mixed and/or additive modelling [43]). The latter ensures a flexible and individual implementation, including an appropriate integration of spatio-temporal autocorrelation [41]. Since SLRMs have been shown to be equivalent to fitting a spatial PPM if sufficiently large *a-priori* regression weights are used [26, 39, 57], the main difference between our SLRM- and PPM-approach lies in the spatio-temporal autocorrelation considered within the PPM, in contrast to the pure spatial SLRM. To point this out, we refer to our PPMs as ‘spatial-temporal point process models’ (ST-PPMs) in the following.

The second method is step-selection models (SSMs) [44], which have been developed from the viewpoint of an individual, in contrast to the population viewpoint adopted in ST-PPMs and related models [45]. SSMs have recently been combined with simultaneous animal movement models estimating movement and resource selection parameters, leading to integrated step selection analysis (iSSM) methods [46]. In the following, we use the expression (i)SSM if we simultaneously refer to both – SSMs and iSSMs. The above-mentioned different viewpoints of ST-PPM and (i)SSM approaches become noticeable e.g., when looking at autocorrelations in the data. In (i)SSM methods, these dependencies are used to create appropriate data stratification and to choose reasonable dummy point locations, whereas in ST-PPMs, these correlations are considered to be a statistical nuisance [45, 46]. It is therefore not surprising that both methods can lead to divergent conclusions [45]. Finally, all four methods (ST-PPMs, SSMs, SLRMs, and iSSMs) can be used not only to test hypotheses, but also to estimate utilization distributions respectively for predictions [8, 38, 46–48].

However, the variety of available statistical tools makes it difficult to select the most appropriate one for analysing a particular set of tracking data and addressing a specific research question. Although partial comparisons between the different approaches have been presented (e.g., [45, 46, 49]), a more comprehensive comparison of the different frequently used methods in terms of their statistical power and type I error rates in interplay with habitat and movement properties has not, to the best of our knowledge, yet been presented.

**Fig. 1:**
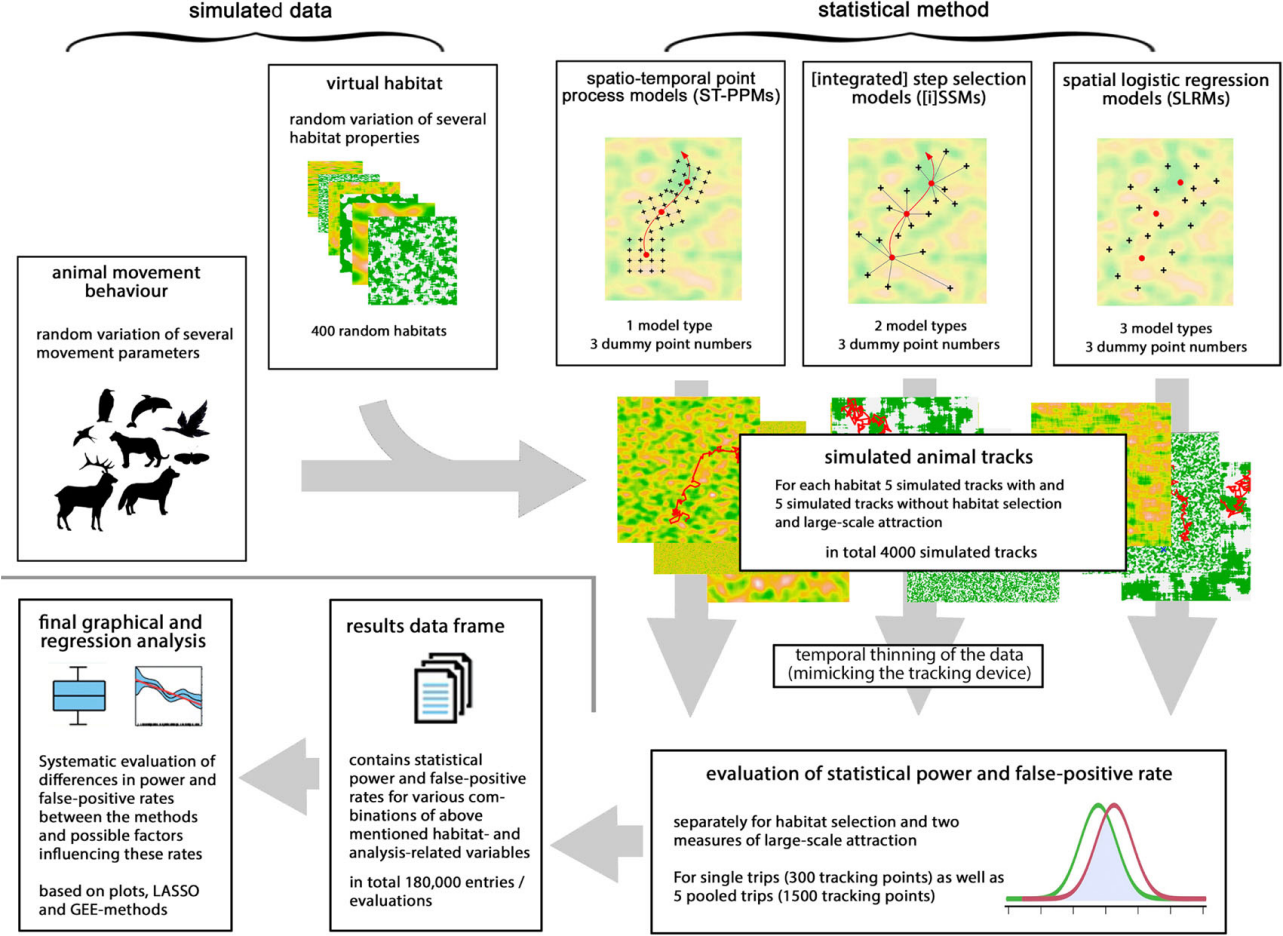
Overview of the presented approach comparing the statistical power and type I error rates of different statistical methods in interplay with simulated habitat and animal movement properties

In the current study, we used simulated data to systematically analyse and compare different statistical approaches (namely SLRMs, ST-PPMs, SSMs, and iSSMs) with respect to local resource/habitat selection, as well as large-scale attraction/avoidance processes. We analysed and compared the statistical powers (i.e., rate of detecting an existent effect) and false-positive rates (type I error rates) in interplay with habitat and animal movement properties (Fig. 1). Based on these results, we provide practical guidance for the conditions under which each of these methods works reliably.

## Methods

### Overview

To cover the potential variety of animal tracking data, we simulated a broad range of reasonable data, resulting from an interplay among various simulated habitat types and animal movement characteristics. The animal movement simulation was conducted on the finest available (pixel) scale, i.e. after a pixel has been occupied, the virtual animal has to decide which of the neighbouring pixels is selected next. In particular (described in more detail e.g. in [8]), this movement is potentially influenced by (1) local habitat attraction; (2) directional persistence; (3) an attraction centre within the virtual landscape having a large-scale effect; and (4) a random component reflecting a certain amount of random walk/unexplained variance. These simulated data are again used below to investigate the statistical power and false-positive (type I error) rates of different frequently used regression methods applied to animal tracking data. A graphical overview of the workflow is given in Fig. 1.

A total of 400 different habitats with randomly varying habitat properties (c.f., “Simulated data” section for more details) were simulated. For each of these habitats, we randomly generated a set of animal movement properties (details below) and used these to generate 10 different animal tracks, five of which were influenced by a randomly selected strength of local habitat attraction representing large-scale attraction (to evaluate the statistical power), and five were not influenced by these attractions (to evaluate false-positive rates). A total of approx. 4000 tracks were therefore available for further analysis and were evaluated using six different regression models belonging to the three main classes: SLRMs, ST-PPMs, and (i)SSMs. In addition, the effect of three different numbers of dummy points (per tracking point) has been analysed, finally leading to approx. 180,000 different model evaluations. The results and covariates were condensed into a summary data frame (c.f., below). Because most of these evaluations again comprised model-selection procedures (c.f., below), approximately 500,000 model fits were performed, some of which required &gt;10 minutes computation time. Parallelized computing using several multi-core computers was therefore used to produce feasible computation times.

Each row of the above summary data frame depicted one unique method–track combination comprising information about (1) the statistical method used, (2) several parameters related to habitat and movement properties (including the respective strengths of the above-mentioned two attraction effects), and (3) the outcome variable (*effect*) defined by the binomial response ‘significant effect detected’ vs. ‘no significant effect detected’ (where the *α*=0.05 significance level has been used). These data were further analysed using appropriate regression model selection techniques, to evaluate and compare the average statistical powers and false-positive rates of the different methods in interplay with the several predictors related to the simulated tracking and environmental data. For the sake of clarity, in the following, variable names starting with *Meth_...* concern the applied statistical method of interest (i.e., a specific variation of a SLRM, ST-PPM, or (i)SSM approach), variables starting with *Hab_...* represent habitat properties, and *σ*... variables define properties related to animal movement or the tracking device. An overview of all considered variables is given in Table 1.

**Table 1:** Possible predictors for statistical power and type I error rates in the context of habitat selection and large-scale attraction based on animal tracking data

Variable name	Explanation
*Hab_auto*	strength of spatial habitat autocorrelation
*Hab_anis*	anisotropy of spatial habitat autocorrelation
*Hab_smooth*	smoothness of transition between habitats
*Hab_type*	continuous or categorical habitat
*σ*_*SD*_	strength of randomness in animal movement
*σ*_*α*_	movement bias towards attraction centre
*σ*_*ω*_	habitat selection strength
*σ*_*ran*_	strength of directional persistence
*σ*_*ran*2_	reducing directional persistence in preferred habitats
*Error*_*spat*_	strength of spatial measurement error
*Meth_SLRM*	spatial logistic regression model, dummy points are randomly generated inside the minimal convex polygon
*Meth_SLRM_s*	as for *SLRM* but with spatial 2D smooth aiming to reduce spatial autocorrelation
*Meth_SLRM_w*	as for *SLRM* but with strong weights assigned to dummy points
*Meth_SSM*	step selection model
*Meth_iSSM*	integrated step selection model including model selection with respect to the autocorrelation terms
*Meth_ST-PPM*	spatio-temporal point process model including model selection with respect to the autocorrelation terms

Predictors related to habitat start with ‘Hab...’, predictors related to animal movement with ‘ *σ*...’, and predictors related to the statistical method applied to simulated tracking data with ‘Meth_...’

### Computation and software

All statistical analyses were performed using the open-source software R [50]. In particular, spatial methods and spatial visualizations were mainly based on the package *raster* [51], and all other statistical plots were performed using *ggplot2* [52]. Additional R-packages and functions are detailed below. All computations were performed using parallelized code on several Intel Xeon Quad Core computers, each with 3.90 GHz and 32 GB RAM.

### Simulated data

#### Habitat data

Habitat raster data were generated based on the *raster()*-function in the R-package *raster* [51], where habitat values (given by the variable *hab*) were randomly assigned (uniformly distributed between 0 and 1) to 1000×1000=10^6^ spatial 2D pixels $\vec X = (X_{1},X_{2})$. Possible spatial autocorrelation (i.e., local spatial clustering of certain habitat types) was quantified by the continuous variable *Hab_auto* ∈[0,1], where a value of 0 represents no autocorrelation (i.e., the *hab* value for each pixel does not depend on the *hab* values in the surroundings) and values of *Hab_auto*&gt;0 represent an increasing isotropic autocorrelation. In contrast, anisotropic autocorrelation was represented by the continuous variable *Hab_anis* ∈[0,1], where a value of 0 represents an isotropic landscape, up to strongly anisotropic landscapes with maximal values of *Hab_anis*=1.0, the latter resulting in habitats stretched along the x-axis. Both types of autocorrelation were generated using a ‘moving window’ approach based on the R-function *focal()* in the *raster* package. Furthermore, the smoothness of the transition between different habitat types was defined by the categorical variable *Hab_smooth*, which can generate sharp, medium, or blurry transitions, also realized in the context of the above-mentioned *focal()*-function. Finally, after generating autocorrelation and transition smoothness, in approximately 50% of cases, the continuous variable *hab* was transformed into a binomial one (based on the threshold of 0.5) where the final type of *hab* was represented by the variable *Hab_type* differentiating between continuous and categorical habitat data. Some examples for simulated categorical habitat data (permutated over the variables *Hab_auto*, *Hab_anis* and *Hab_smooth*) are shown in Fig. S1, and further examples including continuous habitats are presented in Fig. S2 and Fig. 1.

#### Animal movement and resulting tracking data

We simulated movement data using an approach that is strongly related to the ‘stepping-stone’ algorithm, as presented by Avgar et al. [46]. In particular, we simulated 5000 steps of movement on the finest available spatial scale, i.e., on a scale of pixels (in the following termed ‘trip’). For each simulation/trip, the starting location and the location of the attraction centre were chosen randomly within the virtual study area. However, to avoid boundary effects, the starting location was restricted to the 500×500 square in the centre of the area and the attraction centre to the 850×850 square. It was assumed that the boundary was never reached by the virtual track, and boundary-related bias could thus be excluded. For each simulated time step *t* with location $\vec X_{t} = (X_{1},X_{2})$, the probability of choosing the neighbouring pixel $\vec Y = \vec X_{t +1}$ out of the eight nearest neighbours $ \vec Z \in \mathcal {N}$ as the next point was given by

$$\begin{array}{@{}rcl@{}} P\left(X_{t+1}=\vec Y \right) = \frac{\mathcal F(\vec Y)}{\sum_{\vec Z \in \mathcal{N}} \mathcal F(\vec Z)}, \end{array} $$

with

$$ {\displaystyle \begin{array}{lcrr}\mathcal{F}\left(\overrightarrow{Y}\right)=\exp \left(\right.N\left(0,{\sigma}_{SD}\right)& +& {\sigma}_{\omega}\cdotp hab\left(\overrightarrow{Y}\right)-\mu \left\Vert \overrightarrow{Y}-{\overrightarrow{X}}_t\right\Vert & \\ {}& & \kern1em -{\sigma}_{\alpha}\cdotp {\alpha}_{att}& \\ {}& -& f\left({\sigma}_{ran},{\sigma}_{\mathit{\operatorname{ran}}2}\right)\cdotp {\alpha}_{pers}\left)\right.,& \end{array}} $$

where a more detailed motivation of the general structure of this stepping stone algorithm is given by Avgar et al. [46]. Here, *N*(0,*σ*_*SD*_) represents a normally distributed random component in animal movement (where new values are drawn for each evaluation of $\mathcal F(.)$), i.e., scaling the strength of random movement vs. directed/biased movement in the animal path, quantified by the movement standard deviation *σ*_*SD*_∈[0,2.5]. We want to point out that *σ*_*SD*_ as well as all following parameters denoted with *σ* were fixed parameters for each particular simulation, and were varied only across different simulation scenarios (c.f., overview at the beginning of the “Methods” section). *σ*_*ω*_∈[0,1.0] quantifies the strength of resource selection, while the term $\mu || \vec Y - \vec X_{t}||$ with *μ*=1.8 penalizes larger Euclidean distances to the 4 of the 8 neighbouring pixels within a rectangular grid. Furthermore, *σ*_*α*_∈[0,0.1] penalizes angular deviations *α*_*att*_ from the direct path between $\vec Z$ and the attraction centre (and thus introduces directional bias towards the centre, ‘biased random walk’ [8]), and *f*(*σ*_*ran*_,*σ*_*ran*2_) finally penalizes angular deviations *α*_*pers*_ from the direction of the foregoing movement step via the two constant parameters *σ*_*ran*_ and *σ*_*ran*2_, thus leading to directional persistence (‘correlated random walk’ [8]). Notably, $f(\sigma _{ran},\sigma _{ran2}) = \sigma _{ran}/\big (1 + \sigma _{ran2} \cdot \ {\sum _{\vec Z \in \mathcal {N}} hab(\vec Z)/8)} \big)$ includes the parameter *σ*_*ran*_∈[0,2.5] for the general strength of directional persistence, but also the parameter *σ*_*ran*2_∈[0,1.0] antagonizing this effect if local habitat values (averaged over all neighbours) are high. The latter effect thus induced a less-directed and more-random search behaviour in appropriate habitats. Finally, after generating the animal track, a virtual time *t* was assigned to the locations using equidistant time steps of 1 minute. Some example tracks with varying values for *σ*_*ω*_,*σ*_*α*_ and *σ*_*ran*_ are given in Fig. S2.

The simulated animal movement data at the spatial pixel scale (c.f., previous subsection) were subsequently reduced to a much coarser temporal resolution, mimicking the data collected by a tracking device. In particular, a total of 300 tracking points were selected with equidistant time points between the tracking points. After selection of the spatio-temporal subset (‘tracking data’) from the raw animal movement data, as described above, spatial measurement error was added, quantified by the parameter *Error*_*spat*_∈[0,3] depicting the standard deviation of a normally distributed random error separately added to each point and coordinate.

### Statistical analysis of simulated animal tracks

As noted in the Introduction, we applied and compared different variations of statistical models of the most frequently used classes, namely SLRMs, ST-PPMs, SSMs, and iSSMs, based on the analysis of simulated animal tracking data. To obtain optimal comparability, several steps were uniformly applied. First, the same level of dummy point numbers per tracking point (namely *N*_*dummy*∈{8,80,230}) were used in all models. Second, central predictors were entered in all models in a similar manner: *hab* as a linear predictor for habitat selection studies, and *attrac*_*dist* for the evaluation of large-scale attraction measuring the Euclidean distance to the attraction centre. As an alternative to *attrac*_*dist*, the variable *attrac*_*ang* was used, calculating the cosine of the angular deviation from a straight line between the previous tracking data point and the attraction centre, thus representing the directional bias towards the centre. *attrac*_*ang* was not used for SLRMs, because a pure spatial approach does not allow the consideration of angular deviations depending on the temporal order of tracking points.

#### Spatial logistic regression models

SLRMs were implemented using the gam() function in the R-package *mgcv* [53] with a binomial error distribution and logit-link function, where true tracking points served as presence points in the outcome variable, and dummy points were treated as true absences. In particular, dummy points were randomly chosen within the minimum convex polygon (MCP) around the tracking data (calculated by the *mcp()*-function from the *sp*-package [54]) depicting a simple and frequently used approach to estimate home ranges (e.g., [55, 56]) and leading to the model *Meth*_*SLRM*. To account for possible spatial autocorrelation, we optionally added a spatial 2D thin plate regression spline *s*(*X*_1_,*X*_2_) to the predictor (where the optimal number of knots was estimated based on generalized cross-validation [53]), leading to the model *Meth*_*SLRM*_*s*. Finally, as proposed previously [43, 57], we also assigned large *a-priori* regression weights (*W*=1000) to all available points with used points assigned a weight of 1 leading to an ‘infinitely weighted logistic regression model’ [57], in the following termed *Meth*_*SLRM*_*w*. As demonstrated by Fithian and Hastie [57], for *W*→*∞* the SLRM likelihood converges to the likelihood of an inhomogeneous Poisson process. Thus, as mentioned above, the main difference between the SLRMs and the ST-PPMs (c.f., below) lies in the additional consideration of temporal autocorrelation in the PPMs.

#### (Integrated) step selection models

SSM and iSSM analyses relied on conditional logistic regression functions (*fit*_*ssf*() respectively *fit*_*issf*()), as provided by the R-package *amt* [47]. In particular, data were stratified by the time points of tracking points (‘used steps’) and temporally associated dummy points (‘available steps’) [44, 46]. Dummy points were generated based on step lengths and turning angles, assuming a gamma and a von Mises distribution [46, 47]. The iSSM approach differs from the SSM approach by additionally considering fixed-effect predictors related to different movement characteristics [38, 47]. For iSSMs, we therefore performed a model selection step based on the Akaike information criterion (AIC) [58] comparing five different models reflecting all existing combinations of main effects and interactions in relation to the different autocorrelation terms, namely the logarithm of the spatial distance to the forgoing tracking point *log*(*d*_*x*_), and cosine of the turning angle *cos*(*ta*) [47] (due to equidistant time steps, the temporal distance to the foregoing step was not considered). A list of corresponding predictor combinations is given in Table 2. The final model selected from this procedure was termed *Meth_iSSM*, the SSM model was termed *Meth_SSM*.

**Table 2:** Different predictor combinations compared during AIC-based iSSM and ST-PPM selection

iSSM predictors	ST-PPM predictors	
*hab*	*hab*	
*hab*+*log*(*d*_*x*_)	*hab*+*s*(*log*(*d*_*x*_))	
*hab*+*cos*(*ta*)	*hab*+*s*(*cos*(*ta*))	
*hab*+*log*(*d*_*x*_)+*cos*(*ta*)	*hab*+*s*(*log*(*d*_*x*_))+*s*(*cos*(*ta*))	
*hab*+*log*(*d*_*x*_)+*cos*(*ta*) + *log*(*d*_*x*_):*cos*(*ta*)	*hab*+*te*(*log*(*d*_*x*_),*cos*(*ta*))	

*s*(.) defines a cubic regression spline, *te*(.) a tensor product spline [53], *ta* is the turning angle, and *d*_*x*_ the spatial distance to the foregoing tracking point

#### Spatio-temporal point process models

The applied ST-PPMs mainly relied on the approach of Johnson et al. [41]. In particular, we approximated the spatio-temporal PPM likelihood by the expression

$$\begin{array}{@{}rcl@{}} \sum_{j,k} w_{jk} \left(u_{jk} ln\left(\lambda(t_{j},\vec X_{jk})\right) - \lambda(t_{j},\vec X_{jk})\right)  \end{array} $$

with point process intensity *λ*() depending on time points *t*_*j*_ and spatial 2D points $\vec X_{jk}$. Furthermore, *u*_*jk*_=1/*w*_*jk*_ for tracking points and *u*_*jk*_=0 for dummy points with appropriate quadrature weights *w*_*jk*_ [27, 41], where the index *j* always refers to discrete time points and the index *k* to the discretisation in 2D space. This expression is proportional to a weighted Poisson likelihood with weights *w*_*jk*_ and observations *u*_*jk*_, such that standard generalized linear/additive modelling (GLM/GAM) software can be used [27, 41]. Dummy point generation was based on the R-package *mvQuad* [59]. In particular, for each tracking point, we created a rectangular grid of dummy points (centred around the previous tracking point, randomly rotated and with side length *l*). The random rotation was introduced to avoid any directional bias. Quadrature weights were here based on the rectangle rule, outperforming several other possible quadrature weights with respect to maximal ST-PPM statistical power, while showing type I error rates at or below the nominal level (results not shown). However, the optimal spatial extent of the dummy point grid in ST-PPMs is a priori not clear, in contrast to (i)SSM methods where dummy points represent ‘available steps’ and are thus defined by representative spatial step lengths of the tracking point time series. In contrast, for each time point *t*_*j*_ in ST-PPMs, the grid has to be large enough to cover sufficient area where the conditional intensity surface substantially differs from zero on the one hand, but is small enough to obtain a satisfactory spatial resolution on the other. Here, based on Warton and Shepherd [26], we chose the optimal spatial extent based on the empirically determined convergence of the ST-PPM-likelihood. In particular, we first fitted different ST-PPMs (using 8 dummy points per tracking point) by stepwise increasing the dummy point grid extent *l*=2.5,5.0,7.5,... until the relative change in likelihood was ≤1*%* or changed its sign, leading to *l*_*opt*_. We then fitted three ST-PPMs with 8, 80, and 230 dummy points per tracking point using the optimal spatial extent *l*_*opt*_. Alternatively, the availability at any given time point and thus a reasonable set of quadrature points can be determined based on assumptions related to Brownian motion [41].

All ST-PPMs were fitted based on GAM software [60] using the *bam()*-function in the *mgcv* package [53]. Additive models allow the investigation and formulation of nonlinear relationships between different variables [53, 60], which we used to formulate possibly nonlinear autocorrelation-related predictors within the ST-PPM, extending the linear approach of Johnson et al. [41]. In particular, autocorrelation has been integrated as a Markov process (similar to the spatio-temporal PPM approach of [41]), and considering a possibly nonlinear dependency of the tracking data on step length and step heading (due to equidistant time steps, step duration was not considered here). In particular, optimal models were selected based on the AIC (with AIC calculations based on the ST-PPM-likelihood approximation and not on the standard AIC provided by the *mgcv* package), comparing several combinations of these variables (corresponding formulas are given in Table 2).

### Final comparative analysis of model performance

As explained in the introduction to the “Methods” section, the final data for comparing the different statistical methods can be separated into six different data frames: two for analysing habitat selection, two for the analysis of large-scale attraction using a distance-based measure, and two for evaluating large-scale attraction based on an angular measure. Notably, in each case, the two data frames were given by (1) evaluated tracking data partially driven by an existing underlying attraction effect (for evaluating statistical power) or (2) with no underlying effect (for evaluating false-positive rates). In all cases, the binomial variable *effect* (‘detected significant effect’ vs. ‘no significant effect detected’) was used as the outcome variable for further analyses – as described in more detail below (c.f. also Fig. 1).

#### Analysis of single trips

To compare the average statistical power and respective type I error rates between the different statistical methods applied to single trips, we first averaged the binomial outcome variable *effect* for each five trips with similar habitat- and movement-specific covariates. This step was done in order to appropriately account for the hierarchical data structure. We then evaluated these data graphically based on averages for each combination of method and dummy point number, with confidence intervals calculated based on simple bootstrapping by resampling the averaged *effect*-values with replacement and calculating confidence intervals based on appropriate quantiles [61].

Investigation of method-specific power and type I errors in interplay with several habitat and movement-related properties resulted in a high number of possible predictors based on the variables presented in Table 1, because not only the main effects, but also the interaction terms between method- and habitat-/tracking-related variables, were considered. Since analysed data are not completely independent, during the following regression analysis we account for this by using generalised estimating equation (GEE) approaches (more details on this approach are given below). First, we were particularly interested in the factors influencing the statistical power of the different methods in a similar way. In the first GEE, we therefore only investigated the main effects of all variables presented in Table 1. We then investigated the factors driving the differences in power between the different methods. Here, main effects were augmented in a second GEE with interaction terms between model-related and non-model-related variables, with interaction terms representing conditions in which the different methods performed differently.

To handle the large number of possible predictors, we applied an efficient model selection technique before final regression analysis, namely the ‘least absolute shrinkage and selection operator’ (LASSO) technique [62, 63] (based on the R-package *glmnet* [64]). This technique is known to perform reliable model selection even if predictor numbers are high [4]. Notably, we used logistic regression models within the LASSO procedure (directly applied to the binomial response variable *effect*) and combined them with cross-validation [65]. The latter was used to choose a more parsimonious model based on minimizing prediction error associated with independent data [66].

For the final regression analysis, GEE models [67] with an ‘exchangeable correlation structure’ [2] (clustered within the landscape-ID) and a binomial probability distribution were applied to the pre-selected predictors, realized with the *geeglm()-*function in the R-package *geepack* [68]. We favoured GEEs over logistic generalized linear mixed models (GLMMs [2, 69, 70]), because residuals related to the trip IDs (used as a random intercept) strongly violated the normality assumption in GLMMs. Here, GEEs are known to be much more robust against miss-specification in correlation structures [2]. Furthermore, our focus was on describing average patterns across the set of simulations/tracks rather than deriving track-specific interpretations, which also suggested that GEEs were more suitable than GLMMs [71]. Notably, the preceding LASSO step meant that the final GEE-based results did not follow from an a priori model with appropriate type I error control, given that the predictors had been pre-selected. To compensate for this increased risk of type I errors, we applied a Bonferroni correction to the significance level of *α*=0.05 (i.e., we considered *α*/*m* instead, where *m* is the number of predictors used in the LASSO approach).

#### Analysis of multiple trips/animals

Because most modern-day GPS data sets can include thousands of observations, possibly representing multiple trips and/or animals, we additionally evaluated the statistical power and type I error rates for the combined analysis of five trips (i.e., 1500 tracking points) with similar underlying movement- and habitat-related covariates. In particular, we did not analyse the 1500 tracking points directly within one single ST-PPM, SLRM and (i)SSM, but rather further analysed the regression coefficients resulting from the analysis of the single trips (c.f., above). In particular, we analysed each 5 regression coefficients resulting from the application of one of the investigated methods to the above mentioned 5 correlated trips by using linear regression models (LMs). In more detail, we tested whether the mean coefficient was significantly different from 0, summarizing the result of this test as an appropriate binomial variable – ‘detected significant effect’ vs. ‘no significant effect detected’ – for further analysis of power and type I error rates. We chose this approach over direct analysis of combined tracking data from the five trips (e.g., via mixed modelling) because the computation times, particularly of our ST-PPM approach, were beyond the feasible times for 1500 tracking points and high dummy point numbers, even using parallel computing (c.f., Fig. 3). The analysis of statistical power and type I error rates for the multiple-trip analysis was also carried out based on graphical analysis, with confidence intervals again based on simple bootstrapping methods.

#### Evaluation of computation time

All the above analyses were performed based on relatively small numbers of tracking points *N*=300 for analysis of single trips, or 1500 points for the analysis of multiple trips. In the latter case however, as described above, regression methods were not applied directly to the raw tracking data but using estimated regression coefficients from the different trips. With respect to real tracking data sets, the temporal resolution and thus total amount of tracking data is constantly increasing, e.g., due to continuing technical developments. It is therefore of interest to understand how the computation times of the different methods change with large increases in the amount of tracking data. We therefore analysed the impact of tracking-data size on computing time by generating animal trips, largely as described above (c.f. also Fig. 1). However, we varied the size of the simulated tracks for raw tracking data sizes of *N*=500 to *N*=32,000 steps, with resulting tracking data sizes (after temporal thinning) of *N*=50 to 3200 tracking points. Furthermore, we used 80 dummy points per tracking point (i.e., total size of analysed data was 4050 to 259,200 points), and all other variables (related to habitat and movement properties) were again varied randomly. Here, model selection (for ST-PPMs and iSSMs) was not applied, and the formulas presented in the last row of Table 2 were used.

## Results

**Fig. 2:**
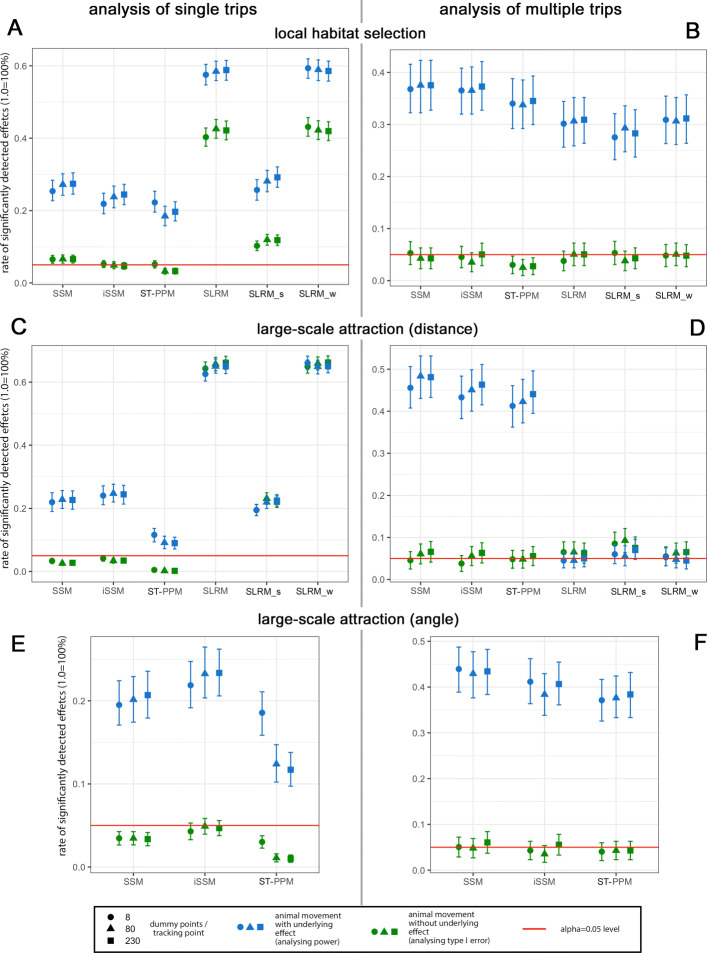
Average comparative performances of different models inferring habitat selection and two different measures of large-scale attraction. Average statistical power using 8 dummy points per tracking point, filled circles; using 80 dummy points, triangles; and using 230 dummy points, squares. Green symbols indicate statistical power (i.e., analysing animal movement with underlying attraction effects) and blue symbols depict type I error rates (i.e., analysing movement without attractions). Error bars represent 95% confidence intervals based on bootstrapping

Figure 2 shows the average statistical power and type I error rates separately for the different statistical methods, the three different measures of interest (habitat selection, large-scale attraction based on distance measure, and large-scale attraction based on angular measure), and separately for the analysis of single vs. multiple trips. Additionally, all evaluations were performed separately using three different numbers of dummy points per tracking point (8, 80, and 230).

**Fig. 3:**
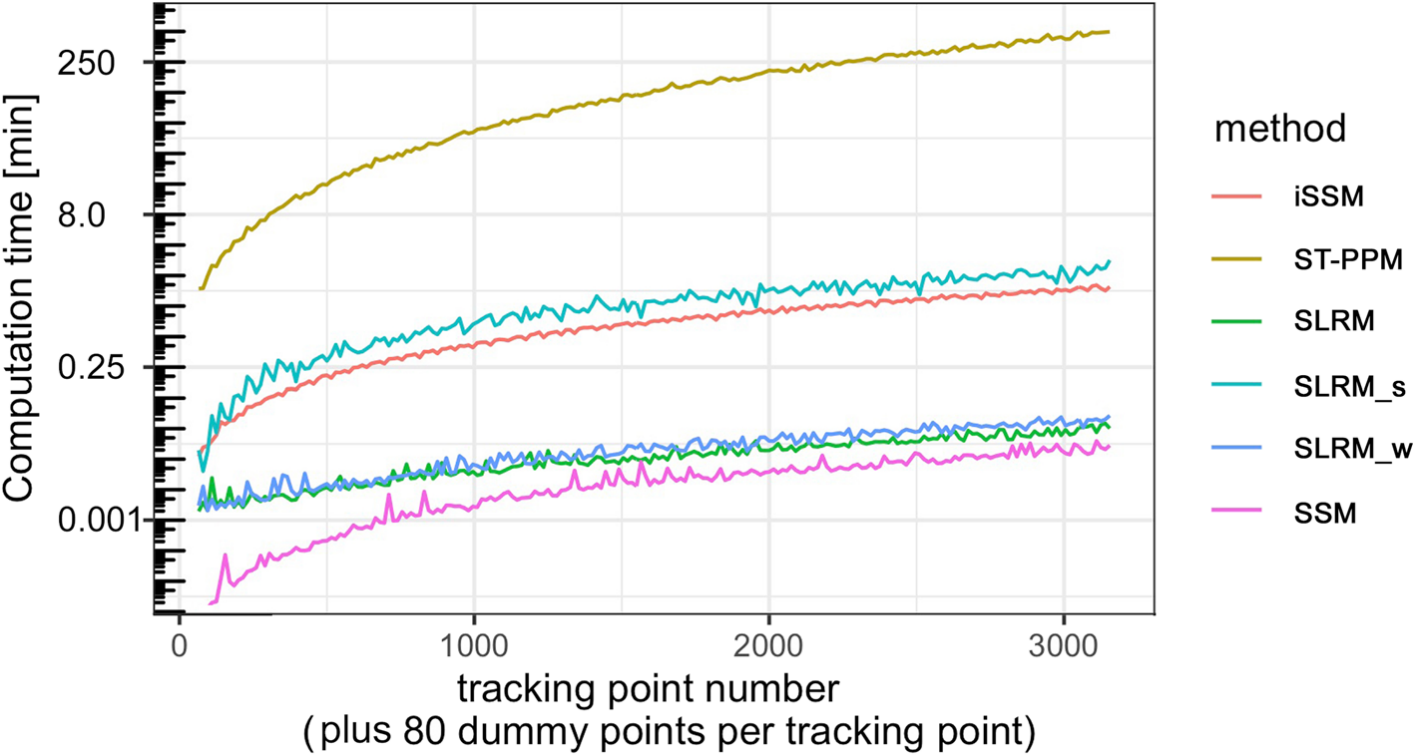
Non-parallel computation times for dummy point generation and model fit for all investigated models, in relation to total number of tracking points. The y-axis shows log-transformed values

### Dummy point numbers

Figure 2 showed that the influences of the considered dummy point numbers on the statistical power and type I error rates were minor: in most cases, increasing *N*_*Dummy*=8 to 80 only resulted in a slight relative increase in statistical power, whereas a further increase from *N*_*Dummy*=80 to 230 resulted in no further improvement in most cases.

### Habitat selection

The average power and type I error rates with respect to the detection of local habitat selection for single and multiple trips are shown in Fig. 2A and B, respectively.

#### Average performance

Notably, all the SLRM-based approaches showed strongly upward-biased type I error rates. Indeed, the model *s_SLRM* showed the smallest (but still too large) type I error rates here, because spatial autocorrelation was reduced by the use of a spatial 2D-regression spline in the predictors. SSMs also showed inflated type I error rates, but only slightly above the nominal level. In contrast, the ST-PPM and iSSM approaches showed type I errors at or below the nominal level, with the iSSM method having a slightly higher average statistical power than the ST-PPM method. Overall, the analysis of multiple trips with respect to local habitat selection (Fig. 2B) was much more consistent, with all methods showing type I error rates at or below the nominal level, and statistical powers ranging from nearly 40% in (i)SSM approaches to approx. 30% in SLRM approaches.

#### Interplay between method and tracking-data properties

To analyse the interplay between the different statistical methods and variables related to environmental and movement properties (restricted to the analysis of single trips), we first restricted the final data frame for the power evaluation to the ST-PPM and iSSM methods (based on 80 dummy points per tracking point), because only these methods showed type I error rates at or below the nominal level (c.f., previous subsection and Fig. 2). As described in more detail in “Final comparative analysis of model performance” section, we then applied a LASSO-based model selection in combination with cross-validation to the data frame to select a set of promising predictors, which were further analysed in a third step using appropriate GEEs. All the significant main effects (with respect to the Bonferroni-corrected *α*-level) and the significant interaction terms (below the double line) are presented in Table 3.

**Table 3:** Significant main effects (above the double line) and interaction terms (below the double line) driving the statistical power in habitat selection studies

Parameter name	Estimate	SE	p
Hab_auto	-0.02669	0.00537	&lt;0.0001
Hab_anis	-0.85126	0.29533	0.00395
Hab_type	-1.27360	0.16523	&lt;0.0001
*σ*_*SD*_	-0.28153	0.03195	&lt;0.0001
*σ*_*ω*_	3.07303	0.31267	&lt;0.0001
Meth_iSSM	0.41505	0.05467	&lt;0.0001
Hab_type:Meth_iSSM	0.69831	0.11372	&lt;0.0001

Results are based on GEE analyses in combination with LASSO-based model selection. Only iSSM and ST-PPM methods were considered because all the other methods showed inflated type I error rates

In accordance with “Average performance” section, the main effects in Table 3 showed that the iSSM had a higher statistical power than the ST-PPM, on average (the latter served as the baseline category in the model-related predictor variable *Meth*). Furthermore, the strength of local habitat attraction (*σ*_*ω*_) strongly positively influenced the power in both methods, as expected. Furthermore, the average power was lower in categorical (*Hab*_*type*) than in continuous habitats (the latter being the baseline-category). This observation generally suggests that categorical habitats provide less local information on animal–habitat interactions than continuously graded variables, affecting the power of the statistical method [72]. Increasingly strong habitat autocorrelation (*Hab*_*auto*) or anisotropy (*Hab*_*anis*) and strength of randomness during animal movement (*σ*_*S*_*D*) decreased the power, all of which were intuitively expected. Finally, the interaction term in Table 3 revealed that the power of iSSMs coped particularly well with categorical habitats, compared with ST-PPMs. Indeed, restricting the power-related data frame during GEE analysis to continuous habitats demonstrated similar powers of ST-PPMs and iSSMs (28% vs. 30%, respectively), whereas the difference was much more pronounced for categorical habitats (9% vs. 17%, respectively).

### Large-scale attraction

The average power and type I error rates with respect to the detection of large-scale attraction are shown in Fig. 2C–F, including the results related to distance to the attraction centre (Fig. 2C, D), and the results based on the evaluation of angular deviations from a straight line between the previous tracking point and the attraction centre (Fig. 2E, F). Due to a lack of information with respect to data chronology, the latter approach cannot be applied to SLRMs. Results for single trips are shown in Fig. 2C, E and results for multiple trips in Fig. 2D, F.

#### Average performance

With respect to the evaluation of distance to the attraction centre (Fig. 2C), SLRMs showed type I error rates far above the nominal level and nearly indistinguishable from the values of statistical power. In contrast, for both considered measures (Fig. 2C, E), type I error rates were nominal for SSM and iSSM approaches and below the nominal level for ST-PPM approaches. The statistical power was also qualitatively and quantitatively comparable for both measures: the iSSM method had higher power, followed by SSM and ST-PPM. When analysing multiple trips, SLRMs showed a strongly reduced power, with power rates again very close to the type I error rates (Fig. 2D). In contrast, the ST-PPM and (i)SSM approaches showed large-scale attraction type I error rates at or below the nominal level. The average power differed only slightly between the methods, being higher for the SSM methods.

#### Interplay between method and tracking-data properties

Similar to the approach for the analysis of habitat selection, we applied a LASSO approach combined with cross-validation and subsequent GEE analysis separately to (1) data resulting from the distance-based large-scale measure vs. the angular large-scale measure, and (2) for analysing main effects alone vs. main effects plus interaction terms between the methods and the habitat- and movement-related variables. All the significant terms (with respect to the Bonferroni-corrected *α*-level) are presented in Tables 4-5.

**Table 4:** Significant main effects driving the statistical power during large-scale attraction studies using a distance-based measure

Parameter name	Estimate	SE	p
*σ*_*SD*_	-0.46437	0.03097	&lt;0.0001
*σ*_*α*_	42.48088	2.85130	&lt;0.0001
Meth_iSSM	1.69953	0.09229	&lt;0.0001
Meth_SSM	1.53532	0.09036	&lt;0.0001

Significant interaction terms have not been obtained here. Results are based on GEE analyses in combination with LASSO-based model selection. Only the SSM, iSSM, and ST-PPM methods were considered because SLRM methods showed inflated type I error rates

**Table 5:** Significant main effects (above the double line) and interaction terms (below the double line) driving the statistical power during large-scale attraction studies using a angle-based measure

Parameter name	Estimate	SE	p
*σ*_*SD*_	-0.46400	0.03078	&lt;0.0001
*σ*_*α*_	42.01831	2.86862	&lt;0.0001
Meth_iSSM	1.09424	0.06964	&lt;0.0001
Meth_SSM	0.81672	0.06702	&lt;0.0001
*σ*_*ran*_:Meth_SSM	0.27923	0.09410	0.00300

Results are based on GEE analyses in combination with LASSO-based model selection. Only the SSM, iSSM, and ST-PPM methods were considered because SLRM methods showed inflated type I error rates

In accordance with the corresponding plots (Fig. 2C, E), the main effects in Tables 4-5 revealed that the SSM and iSSM showed increased power for both measures on average, compared with the ST-PPM. Furthermore, the power strongly increased with increasing large-scale attraction *σ*_*α*_ and decreased (similar to the power in habitat selection) with increasing randomness in animal movement, as expected. No significant interaction terms were observed with respect to the distance-based attraction measure. In the context of the angular measure, the interaction term *σ_ran:Meth_SSM* indicated that SSM coped better with strong directional persistence than ST-PPMs.

### Tracking data size and computation time

The systematic evaluation of the computation times of the different methods applied to tracking data sizes ranging from *N*=50 to *N*=3,200 (plus 80 dummy points per tracking point) is shown in Fig. 3. For better comparability, the y-axis is given on the log-scale. The SSM, SLRM, and SLRM_w methods showed fast computation times of less than 1 min, even for several thousand tracking points plus dummy points. In contrast, the iSSM and the SLRM_s methods required several minutes for *N*=3200, whereas the ST-PPM approach needed several hours to compute the maximal data sets.

## Discussion

Our simulation study demonstrated that, during habitat selection analysis of single trips, only the ST-PPM and iSSM approaches showed type I error rates at or below the nominal level, with iSSMs having more statistical power than ST-PPMs. The observation that all methods had a relatively low power to detect habitat selection (e.g., compared to the power in the context of long-range attraction) does most probably not reflect a corresponding potential/difference in reality, but rather from the specific attraction/selection parameter ranges used during our simulations. The relative difference in power between the methods was small if continuous habitats were considered, but much more pronounced for the analysis of categorical habitats. SSM approaches showed slightly inflated type I error rates, which were probably explained by the fact that these methods parameterise the movement kernel without adjusting for habitat selection, which can lead to biased estimators of habitat-selection parameters [73]. iSSMs in contrast use movement characteristics in the linear predictor to reduce this bias [38, 46]. SLRM-based methods showed strongly inflated type I error rates, probably because the spatio-temporal and angular autocorrelations were not considered appropriately within these methods. In particular (and as mentioned above), SLRMs and PPMs are highly related to each other [26, 39, 57], but the neglect of temporal autocorrelation in SLRMs is the most critical difference. We therefore do not recommend using SLRMs to infer habitat selection from tracking data. This approach would only be appropriate if the tracking data were completely uncorrelated. However, in the light of the constantly increasing spatio-temporal resolution of these data, this is unlikely to be the case, and even sparse tracking data will often be at least spatially correlated (due to the existence of home ranges).

When multiple trips were evaluated to infer local habitat selection, the type I error rates and statistical powers were more similar among all methods, and all the type I error rates were at or below the nominal level. However, (i)SSM methods again showed the highest power and SLRM methods the lowest. The decreased type I error rates in the SSM and SLRM approaches (compared with the analysis of single trips) can be explained by the fact that the simultaneous evaluation of regression coefficients from multiple trips only produced significant results if all (or most) of the coefficients were distinctly either above or below zero. This is rarely the case, given that only some of each five regression coefficients (related to similar habitat and movement properties) showed type I errors and may additionally show different signs (because they scatter randomly around zero if no underlying attraction effect is present). In these multiple-trip analyses, iSSM and SSM methods showed the highest powers, on average.

In summary, with respect to local habitat selection, we strongly recommend using iSSM or ST-PPM approaches (e.g., [41, 46, 47]) to avoid inflated type I error rates, especially if the tracking data are spatio-temporally correlated. In particular, iSSM methods appear to be the most appropriate, with high power for the analysis of both single and multiple trips, combined with type I error rates at the nominal level.

When analysing large-scale attraction, the overall picture for the analysis of single trips was similar to that for habitat-selection analysis: all the considered SLRM approaches appeared to be inappropriate (due to either strongly inflated type I errors or low statistical power), and the iSSM (together with the SSM method) showed the highest power, and no inflation of type I errors was detected for SSMs. In contrast, ST-PPMs showed lower power for both large-scale attraction measures (distance-related and angular measure) than (i)SSMs, combined with type I error rates distinctly below the nominal level, particularly for high numbers of dummy points. The latter could indicate a problem with underdispersion in the data [74], which may not be appropriately described by the Poisson distribution used for ST-PPM approximation. GEE analysis indicated that SSMs were better at handling directional persistence (with respect to power) compared with ST-PPMs. Given that this effect was not detected for the strongly related iSSMs, there is no obvious explanation for this correlation, and the corresponding *p*-value was only slightly below the Bonferroni-corrected alpha level, suggesting the detection of a random correlation.

With respect to multiple trips, SLRMs (which can technically only be applied to the distance-based measure for large-scale attraction) also appeared to be inappropriate due to their low statistical power and slightly inflated type I error rates. SSMs, iSSMs, and ST-PPMs showed very similar results, namely high power combined with nominal type I errors. However, SSMs had slightly higher power than iSSMs, which in turn performed slightly better than ST-PPMs. Interestingly, there were no strong qualitative or quantitative differences between the two types for measuring large-scale attraction (namely, distance-based vs. angular deviation), suggesting that both measures were similarly appropriate.

Based on our evaluation of dummy point numbers, we concluded that increasing *N*_*Dummy*=80 to *N*_*Dummy*=230 did not increase the power or decrease the false-positive rate for any of the considered methods, and even using only *N*_*Dummy*=8 only slightly decreased the power and had a minor effect on type I error rates. Required dummy point numbers are however likely to be problem specific, e.g. depending on the extent of habitat heterogeneity, sampling rate, or on movement characteristics. Instead of providing general guidance on *N*_*Dummy*, we rather recommend increasing the number of points until results stabilise. However, our results suggest that even lower numbers (such as *N*_*Dummy*=10) should not necessarily be a cause for concern, e.g. if the total tracking data size is large and it is necessary to restrict the number of dummy points to reduce computation times.

Although ST-PPMs and iSSMs showed similar statistical powers under some circumstances (e.g., habitat selection in continuous habitats), we recommend using iSSMs rather than ST-PPMs for the following reasons: (1) the power of iSSMs is more robust compared with ST-PPMs (the latter performed worse in categorical habitats and for the detection of large-scale attraction); (2) iSSMs do not need the time-consuming initial empirical determination of the optimal spatial extent of the dummy point grid; which is related to the fact (3) that computation times for iSSMs are much shorter than for ST-PPMs, which is especially important in the context of large data sets; and (4) iSSM model implementation is user-friendly and well-documented, using the provided R-package *amt* and/or the worked examples in the Supplemental Appendices of Ref. [38], in contrast to spatio-temporal PPMs, for which there is currently (to the best of our knowledge) no available R-package. In addition, Avgar et al. [46] noted additional advantages of iSSMs over previous methods, including their predictive capacity (e.g., for landscapes different from the landscape used for the model fit), and the ability to derive and parametrize a mechanistic movement model.

The above difference in computation times between ST-PPMs and (i)SSMs may be because the initial empirical procedure to determine the optimal spatial extent of the dummy point grid required several time-consuming model fits. On the other hand, our ST-PPM implementation may have been programmed in a less time-efficient manner compared with the SSM and iSSM codes provided within the well-established R-package. Further advances in non-empirical methods for determining optimal ST-PPM dummy point grid extension, as well as a more time-efficient code for ST-PPMs could therefore strongly reduce the described differences between ST-PPMs and the other approaches. Indeed, time-efficient methods for estimating ST-PPM integrals have recently been discussed by Hooten et al. [8].

Despite the general, practical reasons for recommending iSSMs over ST-PPMs, ST-PPMs may be preferred in certain situations: e.g., ST-PPMs are assumed to be more appropriate for irregularly-spaced observations [41] compared with iSSM approaches, e.g. because the latter require approximately equidistant time steps [46].

## Conclusions

We provide an extensive simulation study comparing the statistical powers and false-positive rates of different statistical methods frequently used to infer local habitat selection or large-scale attraction/avoidance from animal tracking data. We compared different variants of SLRMs with SSMs, iSSMs, and ST-PPMs, and evaluated the power and false-positive rates in interplay with a broad range of simulated habitat and movement properties. Our results suggest approximately 50 dummy points per tracking point is a reasonable order of magnitude for all methods, but even using 10 dummy points per tracking point (e.g. if large tracking data sizes are analysed) will only slightly decrease the power, and type I errors most probably remain at or below the nominal level. With respect to the different methods, SLRMs appear to be inappropriate for analysing autocorrelated tracking data, at least if simple schemes for dummy point selection are applied. SSMs may show slightly increased type I error rates in habitat-selection studies, and only iSSM and ST-PPM approaches showed false-positive rates at or below the nominal level in all our case studies. Here, iSSM approaches showed higher power than ST-PPMs, suggesting that iSSMs represent the most appropriate method for analysing local habitat selection and large-scale attraction/avoidance. Other reasons to prefer iSSMs over PPMs include user-friendly software, greater robustness (e.g., with respect to habitat properties), and much faster computation times. In addition, iSSMs, as a recent extension of SSMs, provide the additional advantage of increased predictive capacity in combination with the derivation of a parametrized mechanistic movement model.

## List of abbreviations

A list of abbreviations used in this study is given in Table 6.

**Table 6:** List of abbreviations used in this study

Abbreviation	Explanation
SLRM	‘spatial logistic regression model’; pure spatial approach for analysing tracking data
ST-PPM	‘spatio-temporal point process model’; spatio-temporal approach for analysing tracking data, developed from an Eulerian point of view
SSM	‘step selection model’ (also called ‘step selection function /SSF’ or ‘step selection analysis / SSA’); spatio-temporal approach for analysing tracking data, developed from a Lagrangian point of view
iSSM	‘integrated step selection model’; integrating the SSM approach with a mechanistic movement model
MCP	‘minimal convex polygon’; minimal convex polygon containing all tracking points, for estimation of the minimum home rage
GLM	‘generalized linear model’; extension of linear regression models for non-normally distributed data
GAM	‘generalized additive model’; extension of GLM to describe non-linear (‘additive’) dependencies
GEE	‘generalized estimation equation’; estimation method for parameters of a GLM with possible unknown correlation between outcomes
GLMM	‘generalized linear mixed model’; extension of GLM to describe outcome correlation with random effects
LASSO	‘least absolute shrinkage and selection operator’; technique for model selection that can especially cope with a high number of possible predictors

## Supplementary Information

**Additional file 1** **Figure S1**: Examples of simulated categorical habitat data permutated over the variables *Hab_auto* (strength of spatial autocorrelation), *Hab_anis* (strength of autocorrelation anisotropy), and *Hab_smooth* (blurry vs. sharp transition between habitat boundaries).

**Additional file 2** **Figure S2**: Examples of simulated animal tracks with different underlying strengths of *σ*_*ω*_ (habitat selection strength), *σ*_*α*_ (strength of bias towards the attraction centre), and *σ*_*ran*_ (strength of directional persistence). Blue point represents location of the attraction centre.

## Data Availability

All data are available within this publication and the supporting material. Declarations
