# Correction to: Analysis of local habitat selection and large-scale attraction/avoidance based on animal tracking data: is there a single best method?

**DOI:** 10.1186/s40462-021-00269-3

**Published:** 2021-06-11

**Authors:** Moritz Mercker, Philipp Schwemmer, Verena Peschko, Leonie Enners, Stefan Garthe

**Affiliations:** 1 Bionum GmbH - Consultants in Biostatistics, Hamburg, Finkenwerder Norderdeich 15 A, Hamburg, Germany; 2 Institute of Applied Mathematics (IAM) Heidelberg University, Im Neuenheimer Feld 205, 69120 Heidelberg, Germany; 3 Research and Technology Centre (FTZ) Kiel University, Hafentörn 1, 25761 Büsum, Germany

**Correction to: Mov Ecol 9, 20 (2021)**

**https://doi.org/10.1186/s40462-021-00260-y**

Following publication of the original article [1], the authors identified an error in the affiliation list, due to a typesetting mistake: affiliations 2 and 3 were interchanged.

In addition, the authors identified an error in the captions for Tables 3 and 5 and in the first paragraph of the ‘Interplay between method and tracking-data properties’ section, due to a typesetting mistake. The text “above the double line” and “below the double line” should have been corrected to “above” and “below”.

The affiliation list has been updated above and the original article [1] has been corrected. The original article [1] has also been corrected with regards to the errors in the captions for Tables 3 and 5 and in the first paragraph of the ‘Interplay between method and tracking-data properties’ section. The publisher apologises to the authors and readers for the inconvenience caused by these typesetting mistakes.
