# Supplementary figures and images for: Analysis of local habitat selection and large-scale attraction/avoidance based on animal tracking data: is there a single best method?
Source: Mov Ecol. 2021 Apr 23;9:20. doi: 10.1186/s40462-021-00260-y (PMC8063450; doi:10.1186/s40462-021-00260-y)

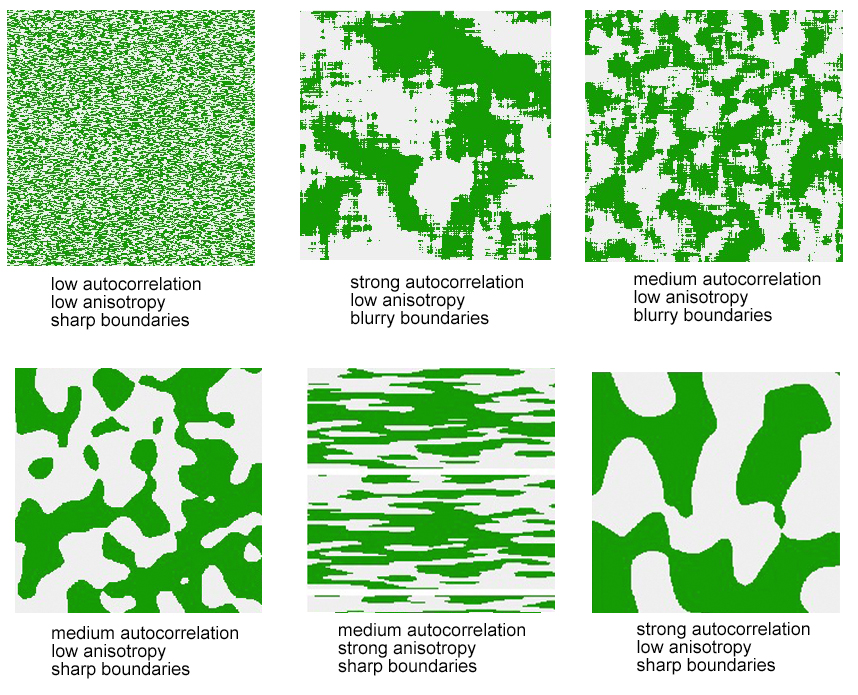

Supplement: Supplementary file 1 — Additional file 1 Figure S1: Examples of simulated categorical habitat data permutated over the variables Hab_auto (strength of spatial autocorrelation), Hab_anis (strength of autocorrelation anisotropy), and Hab_smooth (blurry vs. sharp transition between habitat boundaries). [file 40462_2021_260_MOESM1_ESM.jpg]

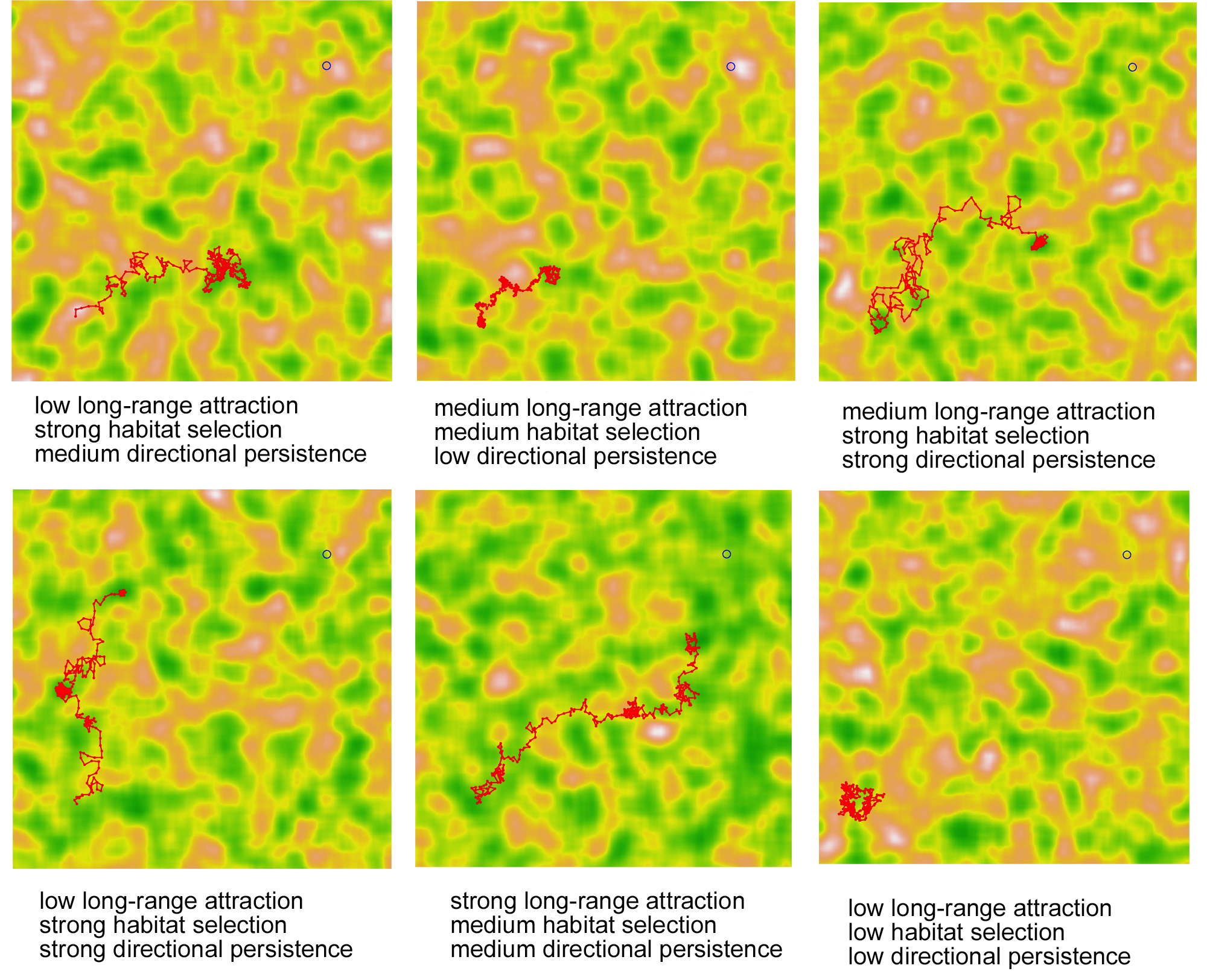

Supplement: Supplementary file 2 — Additional file 2 Figure S2: Examples of simulated animal tracks with different underlying strengths of σω (habitat selection strength), σα (strength of bias towards the attraction centre), and σran (strength of directional persistence). Blue point represents location of the attraction centre. [file 40462_2021_260_MOESM2_ESM.jpg]
